# Differences in change blindness to real-life scenes in adults with autism spectrum conditions

**DOI:** 10.1371/journal.pone.0185120

**Published:** 2017-10-11

**Authors:** Chris Ashwin, Sally Wheelwright, Simon Baron-Cohen

**Affiliations:** 1 Autism Research Centre, Department of Psychiatry, University of Cambridge, Douglas House, Cambridge, United Kingdom; 2 Department of Psychology, University of Bath, Bath, United Kingdom; 3 Department of Cancer Sciences, University of Southampton, Southampton, United Kingdom; 4 Cambridgeshire and Peterborough NHS Foundation Trust, CLASS Clinic, Cambridge, United Kingdom; University of Plymouth, UNITED KINGDOM

## Abstract

People often fail to detect large changes to visual scenes following a brief interruption, an effect known as ‘change blindness’. People with autism spectrum conditions (ASC) have superior attention to detail and better discrimination of targets, and often notice small details that are missed by others. Together these predict people with autism should show enhanced perception of changes in simple change detection paradigms, including reduced change blindness. However, change blindness studies to date have reported mixed results in ASC, which have sometimes included no differences to controls or even enhanced change blindness. Attenuated change blindness has only been reported to date in ASC in children and adolescents, with no study reporting reduced change blindness in adults with ASC. The present study used a change blindness flicker task to investigate the detection of changes in images of everyday life in adults with ASC (n = 22) and controls (n = 22) using a simple change detection task design and full range of original scenes as stimuli. Results showed the adults with ASC had reduced change blindness compared to adult controls for changes to items of marginal interest in scenes, with no group difference for changes to items of central interest. There were no group differences in overall response latencies to correctly detect changes nor in the overall number of missed detections in the experiment. However, the ASC group showed greater missed changes for marginal interest changes of location, showing some evidence of greater change blindness as well. These findings show both reduced change blindness to marginal interest changes in ASC, based on response latencies, as well as greater change blindness to changes of location of marginal interest items, based on detection rates. The findings of reduced change blindness are consistent with clinical reports that people with ASC often notice small changes to less salient items within their environment, and are in-line with theories of enhanced local processing and greater attention to detail in ASC. The findings of lower detection rates for one of the marginal interest conditions may be related to problems in shifting attention or an overly focused attention spotlight.

## Introduction

Change blindness is a phenomenon where people fail to spot even large changes in a scene, when changes occur during a visual disruption [1,2] (Rensink et al., 1997; Simons & Levins, 1997). This effect occurs because we do not retain a full representation of all visual details in our environment from one moment to the next, so attention towards an item is necessary to detect a change in it [3,4] (Rensink, 2000; Rensink et al, 2000). Since the environment contains too much information to process at any one moment, attention allows us to attend selectively to the most important items and to devote less attention to information of less interest.

Autism spectrum conditions (ASC) are characterized by social and communication difficulties, alongside unusually narrow interests and repetitive behaviour [5] (American Psychiatric Association, 2013). Repetitive behaviours in ASC include resistance to change and a need for sameness, where even the smallest changes in the environment or routine may lead to a tantrum until the change is undone to restore the environment to its original state [6,7,8](Baron-Cohen, 2006; Kanner, 1943; Volkmar et al., 2005). The heightened perception of changes to local details in ASC is consistent with experiments reporting atypical sensory processing [9] (Tavassoli et al, 2014), and reports of enhanced visual perception in some [10,11] (Ashwin et al., 2009; Latham et al., 2013) but not all studies [12] (Tavassoli et al., 2011). Enhanced sensory processing may emerge from an imbalance in excitatory and inhibitory cortical activity in ASC [13,14] (Rubenstein & Merzenich, 2003; Vattikuti & Chow, 2010) and/or a steeper gradient of attention [15,16,17] (Robertson et al., 2013a,b,c), which may reflect lower levels of the neurotransmitter GABA [18] (Robertson et al., 2016).

A number of theories emphasize enhanced perception of detail as a central feature of ASC [19,20,21,22,23] (Baron-Cohen et al., 2009; Frith, 2003; Happe, 1999; Mottron et al., 2006; Plaisted et al., 2006), supported by research showing superior performance in ASC compared to controls on the Block Design task [24] (Shah & Frith, 1993), the Embedded Figures Test [25,26] (Jolliffe & Baron-Cohen, 1997; Shah & Frith, 1983), and visual search tasks [27] (Plaisted et al., 1998). These tasks involve the analysis of a shape in terms of its constituent parts, with greater feature detection in ASC attributed to differences in lateral inhibition during visual perception leading to sharper spatial attention [15,16,17] (Robertson et al., 2013a,b,c).

Despite this strong evidence for enhanced attention to detail in ASC, there have been mixed findings regarding the detection of change. One study of change detection in children with and without ASC reported no group differences in the ability to detect changes in objects [28] (Burack et al., 2009). Studies of change blindness have similarly failed to show convincing support for heightened attention to changes in ASC. In fact, a number of studies have reported *greater* change blindness in ASC compared to controls [29,30,31,32] (Fletcher-Watson et al., 2006; Fletcher-Watson et al., 2008; Kikuchi et al., 2009; New et al., 2010), which is inconsistent with theories of enhanced perception of details in ASC. Other change blindness studies have found no differences overall between adults with and without ASC [33] (Loth et al., 2008). In contrast, Smith and Milne [34] (2008) reported reduced change blindness effects in adolescents with ASC using short films where participants had to detect errors of continuity, with group differences being more apparent for items of marginal interest. A similar finding was reported for children with ASC who showed faster detection of changes in images of everyday life than controls, with the effect once again being most evident for items of marginal interest [35] (Fletcher-Watson et al., 2012).

One potential explanation for the mixed findings to date in change detection experiments in people with ASC compared to controls is the differences in methodology used across the studies. In particular, differences in the responses required by participants may have affected previous results in those with ASC, who have difficulties in aspects of executive function (EF) [36,37] (Ozonoff, 1995; Ozonoff et al., 1991). Previous research in change detection using more complex response methods requiring participants to press different buttons throughout the experiment have reported greater change blindness compared to controls [29] (Fletcher-Watson et al., 2006), while another change detection study using the same types of stimuli but with a simpler response involving the participant pressing just one button whenever they detected a change, reported reduced change blindness compared to controls which was particularly evident for changes to items of marginal interest [35] (Fletcher-Watson et al., 2012). However, reduced change blindness using the same types of static images and simple response requirement has not been reported to date in adults with ASC.

The aim of the present study was to investigate the detection of changes to items of central and marginal interest in scenes of everyday life in adults with and without ASC. Based on psychological models of enhanced local perception in ASC we predicted the ASC group would show reduced change blindness versus controls. Furthermore, based on previous reports and evidence for greater attention to items that others find insignificant, we also hypothesized that group differences in change detection would be particularly evident for marginal interest items.

## Materials and methods

### Participants

We recruited 22 adult male participants with ASC (mean age = 32.7 years, SD = 11.7, Range = 19–57; mean full-scale IQ = 123.7, SD = 11.5, Range = 101–142). All the participants with ASC had a diagnosis according to internationally accepted criteria [5,38] (American Psychiatric Association, 2013; WHO, 2008). They were all diagnosed in recognized specialist clinics by a psychiatrist, clinical psychologist, or related medical professional and had registered to take part in research through the lab website (www.autismresearchcentre.com). In addition to receiving a clinical diagnosis, they all completed the Autism-Spectrum Quotient (AQ) as a measure of autistic traits [39] (Baron-Cohen et al., 2001).

We also recruited 22 adult male participants (mean age = 32.1 years, SD = 11.6, Range = 20–61; full-scale IQ = 122.49, SD = 12.8, Range = 92–141) with no history of any psychiatric condition, from the local Cambridge community as a control group. Both groups were matched on age and handedness. All participants completed the 4-item version of the Wechsler Abbreviated Scale of Intelligence (WASI) [40] Wechsler, 1999) as a measure of intelligence and had normal or adjusted to normal vision. The study was approved by the Cambridge Psychology Research Ethics Committee, and everyone who took part gave written informed consent. The research was carried out in compliance with the Helsinki Declaration.

### Materials

Stimuli included 51 colour images of real-life scenes that were 27° wide and 18° high and have been used in previous research studies of change blindness [1,4,41] (Rensink et al., 1997; Rensink et al., 2000; O’Regan et al., 1999). Three of the images were used in initial training trials, and the other 48 of the images were used in the experimental trials. Each image had a modified version containing a single change located somewhere within it. The change was either in the colour, location, or presence/absence of an object or area.

There were two types of changes within the scenes, which were defined in previous research according to the interest of people to look in the area where the changes occurred [1] (Rensink et al., 1997). Half the pictures had changes that occurred in areas of the picture where people typically have a high degree of interest to attend and were referred to as ‘*Central interest*’ changes. The other half of the pictures had changes that occurred in areas of the picture where people typically have a low degree of interest to attend, and were referred to as ‘*Marginal interest*’ changes (for further information about the creation and validation of these images see [1] Rensink et al, 1997). Thus, the experiment itself included 48 pairs of images of real-life scenes. Each pair consisted of an original and a modified version, with 24 of the pairs containing a Central interest change and 24 pairs containing a Marginal interest change. The number of colour, location, and presence/absence changes was matched equally across both types of changes.

### Procedure

Participants were seated at a desk in a quiet room and viewed pictures on a 20 in. computer monitor situated approximately 57 cm in front of them at eye level. The experiment was run on a DELL Inspiron laptop and the program DMDX [42] (Forster & Forster, 2003) was used to present stimuli and record responses, which were made on a specially constructed response box designed to be compatible with both the computer and DMDX program.

Each trial began with a cross-hair in the centre of the screen for 500 ms followed by the presentation of one image displayed for 3000 ms. This was immediately replaced by a blank white screen for 200 ms, and followed by the modified version of the image for 3000 ms. The modified image was replaced by the blank white screen for 200 ms, and this was followed by the original version of the image once again. This pattern was repeated until the change was detected or until the trial timed out, which was 38.2 seconds. The present study included longer display times for the experimental images and the blank white flicker screen in each trial compared to many previous similar experiments because we felt the longer display times would reduce response difficulties for the ASC group, since they often have longer response latencies in experimental tasks compared to controls and have difficulties in EF [36,37] (Ozonoff, 1995; Ozonoff et al., 1991). However, previous research has shown that greater latency display times consistent with those used in the present experiment have little effect on change detection [3,4,42,43] (Rensink, 2000; Rensink et al., 2000; O’Regan et al., 1999; O’Regan et al., 2000).

Whenever participants identified a change they pressed a button with their right index finger to stop the trial. After pressing the button, participants reported the type of change they detected and described the part of the scene they saw changing, to ensure they were not simply guessing. There was rarely an instance where someone in either group reported a change that did not correspond to the change or location in the scene (< 1% overall). Participants then pressed the button to move onto the next trial. There were three training trials at the beginning to ensure participants understood the task before beginning the experiment, and these images used in training did not appear in the experimental trials. The presentation order of images was randomized across participants.

### Statistical analysis

There were two dependent measures: (1) Response Latencies to detect changes on trials where a change was successfully detected, and (2) Detection Rates, which represents the mean number of trials where the change was successfully detected. We did not include the maximum trial time value for trials where no change was detected in order to avoid response biases in the data. All the data was tested for normality and results from Kolmogorov-Smirnov tests revealed the Response Latencies were normally distributed for all conditions across both groups (all p > .05). Results for Detection Rate data revealed that data for 10 out of the 12 variables were not normally distributed (p < .05). Log transformations were done but failed to create data with normal distributions, therefore, non-parametric statistics were carried out to analyse the Detection Rates.

A general linear model ANOVA with repeated measures was performed on response latencies with Interest Type (Central vs. Marginal) and Type of Change (Location vs. Colour vs. Absence) as the within-subject factors and Group (Controls vs. ASC) as the between-subject factor. Post hoc t-tests were run where appropriate, with no corrections. Since the data for Detection Rates was not normally distributed, seven different Mann-Whitney U tests were carried out on the data for each Type of Change within each Interest Type, as well as the overall detection rates in the experiment for each group. Since multiple tests were conducted, a Bonferroni correction was applied by multiplying the probability values of each result by 7 to create new probability values to compare to the standard alpha value of .05.

## Results

There were no significant differences between the groups for age, *t*(42) = 0.58, *p* = .59, or for full-scale IQ, *t*(42) = 0.38, *p* = .70. The results of the AQ scores for the sample with ASC (mean AQ score = 38.2, SD = 6.0) included 88.0% of them scoring at or above the cut-off of 32, which is indicative of a diagnosis of ASC (Baron-Cohen et al., 2001).These scores were very similar to findings from previously published studies using the AQ with ASC samples [39] (Baron-Cohen et al., 2001) with a mean AQ score of 35.8 (SD = 6.5) and 80% of the sample scoring at or above the cut-off score of 32.

### Detection rates

The ASC group detected a mean of 43.1 changes out of the 48 total changes overall in the experiment, representing a detection rate of approximately 90%. The control group detected a mean of 44.2 changes, representing approximately 92% of the total number of changes. Mann-Whitney U tests were done including Bonferroni corrections with a probability value of .007 on the Detection Rates in the experiment and revealed no group differences in detection accuracy rates overall across the whole experiment, U = 159, *p* = .05 There were also no differences between groups for 5 of the experimental conditions (all *p*’s > .007), including all three types of changes within the Central Interest trials and changes of colour and absence/presence within the Marginal Interest trials. The only significant group difference that emerged between groups was for changes in location within the Marginal Interest trials, *U* = 123.5, *p* = .005, with the ASC group (mean detections = 4.5) detecting approximately one fewer change compared to the control group (mean detections = 5.7).

### Response latencies

**Fig 1:**
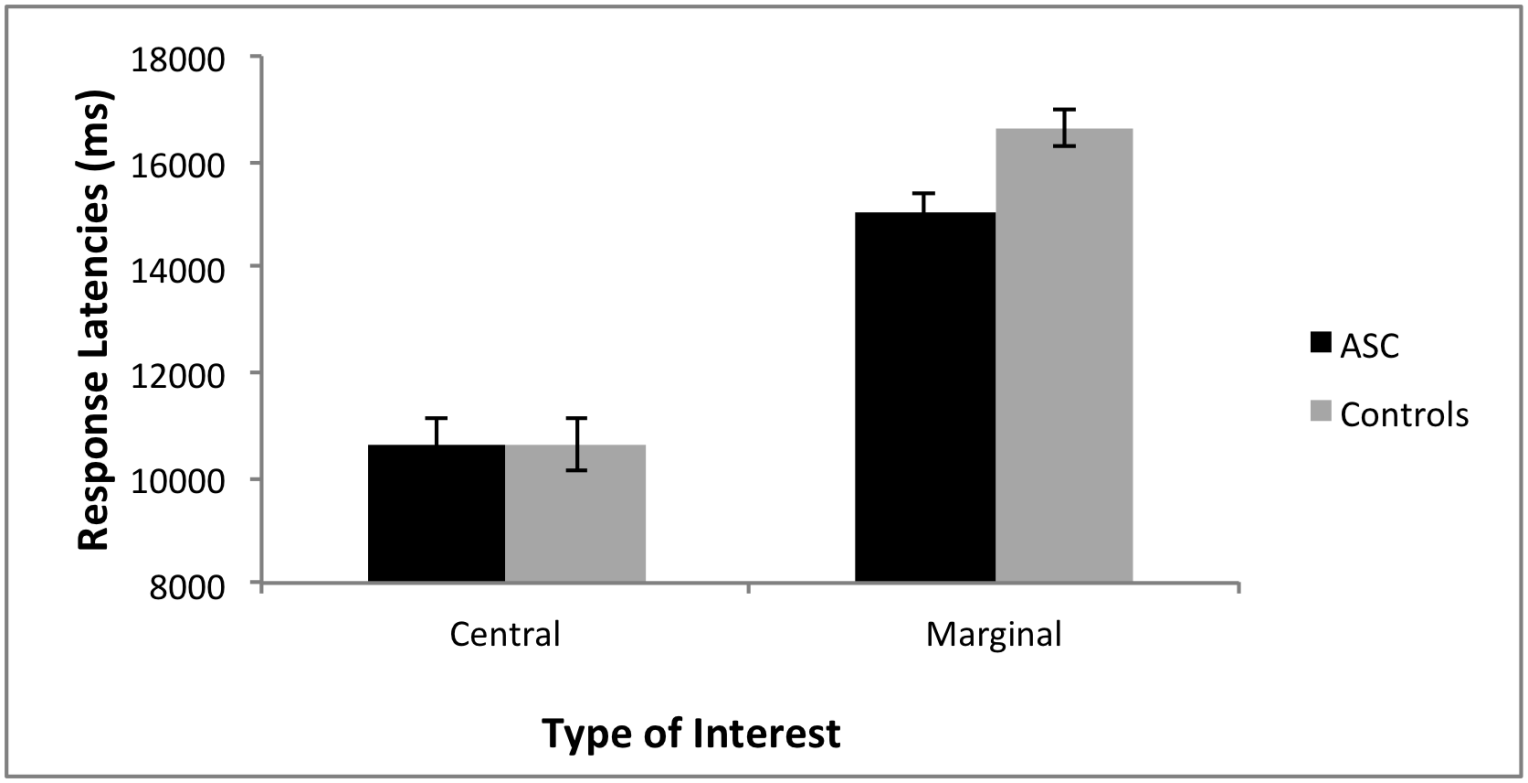
Response latencies for central and marginal interest changes. Mean response latencies and standard errors for both groups to detect changes in central and marginal interest conditions. The ASC group was significantly faster than the control group to detect changes to marginal interest items, with no group differences for changes to central interest items.

The statistics on response latencies for the detected changes revealed there was no main effect of Group, *F*(1, 42) = 1.85, *p* = .18, $\eta_{p}^{2}=0.04$, showing no group differences overall in responses latencies to detect changes. However, there was a main effect of Interest Type, *F*(1, 42) = 275.1, *p* < .000, $\eta_{p}^{2}=0.87$, with participants in both groups detecting the central interest changes quicker than marginal interest changes (Fig 1). This revealed that both groups showed a change blindness effect similar to previous studies.

Importantly, there was an interaction between Group and Interest Type, *F*(1, 42) = 5.1, *p* = .03, $\eta_{p}^{2}=0.11$. Post hoc independent samples t-tests showed there was no difference in response latency to detect Central interest changes for the ASC versus control group, *t*(42) = 0.36, *p* = .97. However the ASC group was significantly faster in detecting Marginal interest changes than the control group, *t*(42) = 2.8, *p* = .008 (Fig 1). Since there was a group difference in Detection Rates for changes of location, a further ANOVA was run without including the response latencies for location changes, and results still showed faster response latencies for the ASC group compared to the control group for detecting Marginal interest changes.

**Fig 2:**
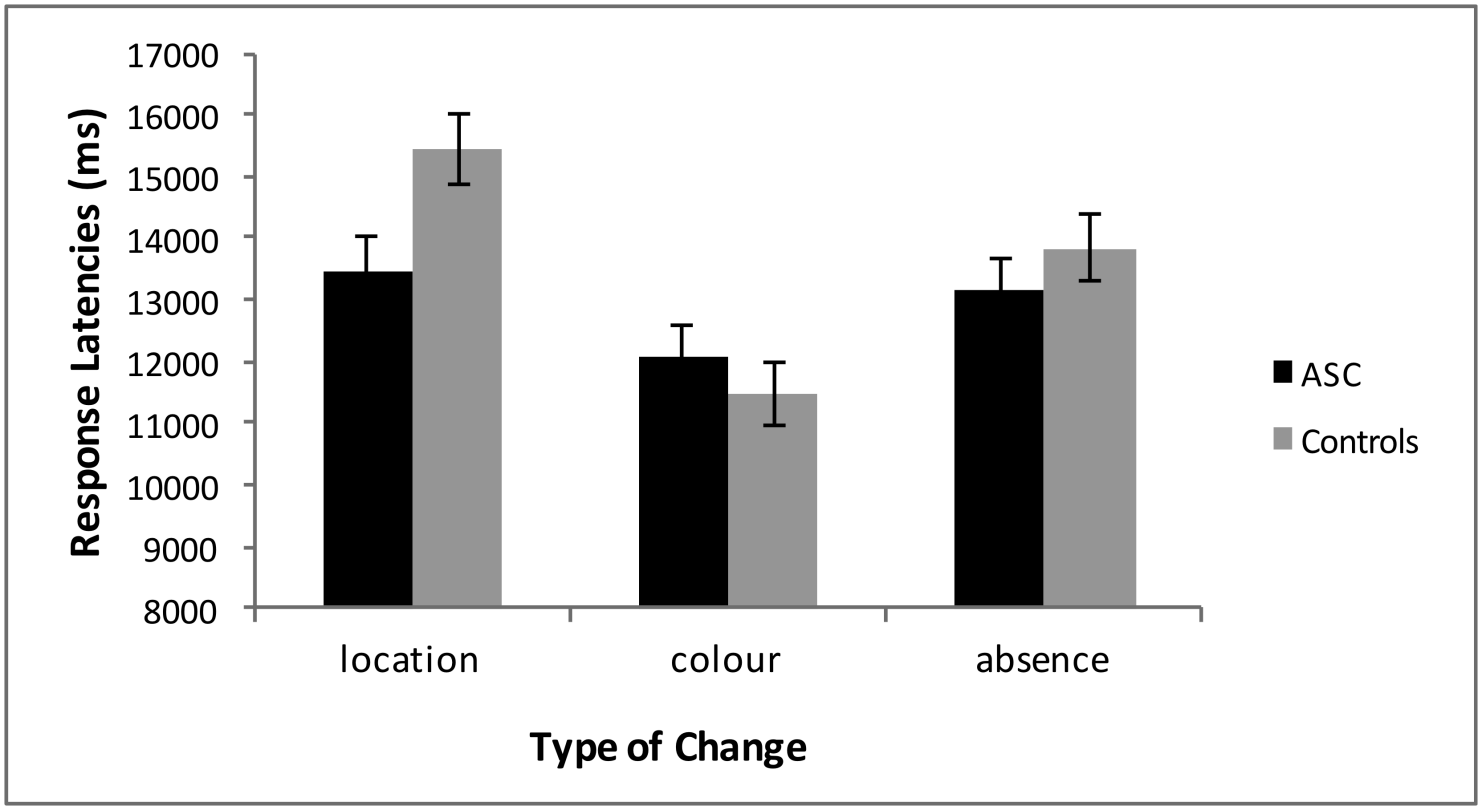
Response latencies for central and marginal interest changes. Mean response latencies and standard errors by both groups for each of the three categories of changes. The ASC group was significantly faster than the control group to detect changes of location, with no group differences to detect changes of colour or absence/presence.

There was also a main effect of Type of Change, *F*(2, 41) = 19.03, *p* < .000, $\eta_{p}^{2}=0.26$. Post hoc t-tests showed that a change in colour was detected faster than in both a change in location (*p* < .000) and a change in absence/presence, (*p* = .001), while no differences were found for response latencies to location compared to absence/presence changes (*p* = .10) (Fig 2).

There was an interaction between Group and Type of Change, *F*(2, 41) = 3.92, *p* = .044, $\eta_{p}^{2}=0.07$. Post hoc independent-samples t-tests between groups showed that there were no differences for changes in colour, *t*(42) = 0.81, *p* = .42, or absence/presence, *t*(42) = 0.88, *p* = .38, but that the ASC group had faster response latencies than controls to detect changes in location *t*(42) = 2.5, *p* = .018 (Fig 2). There was no interaction found between Interest Type, Type of Change, and Group, *F*(2, 41) = 0.11, *p* = .84, $\eta_{p}^{2}=0.01$.

## Discussion

The present findings reveal differences in change blindness in adults with ASC compared to controls. On the one hand, the ASC group showed faster detection of changes to items of marginal interest by adults with ASC compared to control adults, demonstrating reduced change blindness for changes to items within scenes that are typically of lower salience. On the other hand, the ASC group detected approximately one fewer change in location compared to controls of within the marginal interest item changes, although the groups did not differ overall in the number of changes they detected and all participants performed quite well in the task (both groups at 90%+ detection rate). Together, the results demonstrate that people with autism attend to the world in a different way compared to controls, particularly towards items in scenes that are typically of less interest to others.

The present results are consistent with greater attention to small changes in the environment in people with ASC, as the detection of changes in change blindness paradigms requires attention to be focused on the location of the change (Simons & Rensink, 2005). Similar findings for reduced change blindness to marginal interest items in children and adolescents with ASC have been reported using images of scenes and short films [34,35] (Fletcher-Watson et al., 2012; Smith & Milne, 2008). Numerous clinical and anecdotal reports emphasize a local processing bias in ASC and a focus on small details around them that often appear insignificant to others [34,44,45,46] (Baron-Cohen, 2003; Cléry et al., 2013b; Fein et al., 1979; Smith & Milne, 2008). The enhanced sensitivity to changes may help explain the tantrums seen in children with ASC resulting from even small changes in their environment, with insistence on sameness being a central feature of ASC. If people with ASC have heightened awareness of small changes, this may overload sensory and perceptual processing resources and cause over-arousal [34,47,48] (Belmonte, 2000; Belmonte & Yurgelun-Todd Smith & Milne, 2008). The resistance to change and repetitive behavior seen in ASC might be adaptive, to help reduce the excess sensory and perceptual processing and the resulting arousal in ASC from small changes in their surroundings.

The finding of attenuated change blindness in ASC are also consistent with models and research showing enhanced perception of details alongside reduced processing of the context [19–26] (Baron-Cohen et al., 2009; Frith, 2003; Happe, 1999; Jolliffe & Baron-Cohen, 1997; Mottron et al., 2006; Plaisted et al., 1998; Plaisted et al., 2006; Shah & Frith, 1993; Shah & Frith, 1983). One recent study found children with ASC are faster on visual search tasks than typical children, and further showing to be as fast as typical adults [49] (Hagmann et al., 2016). The greater ability by those with ASC to detect certain changes in the present study is even more remarkable considering the task required all participants to intentionally look for a change between the images. The present results are in-line with other findings of reduced change blindness to marginal items in children and adolescents with ASC [34,35] (Fletcher-Watson et al., 2012; Smith & Milne, 2008), and extend these findings to include adults with ASC.

The detection of change relies on focused attention to the changing item, and people with ASC are said to have more focused attention compared to controls [50–52] (Lovaas & Schreibman, 1971; Lovaas et al, 1971; Rincover & Ducharme, 1987). An attentional focus with a sharper gradient around it was recently demonstrated in ASC compared to controls [15] (Robertson et al., 2013a). An overly focused attention may enhance change detection by allocating attentional resources to discrete locations, which facilitates the processing of details within the focus and produces less interference from distracting items around it. Greater attention to the marginal interest changes may also be due to a greater perceptual capacity compared to controls. Enhanced perceptual capacity has been demonstrated in ASC using response-competition paradigms and visual detection tasks [53,54] (Remington et al., 2009; Remington et al., 2012), and is thought to underlie findings of reduced inattentional blindness in ASC [55] (Swettenham et al., 2014). It is proposed that greater perceptual capacity is related to change blindness findings [55] (Swettenham et al., 2014). When change blindness conditions involve a low load, with load referring to the perceptual requirements of a task, then group differences are less likely to be found according to this view because both groups have adequate perceptual resources available. But under conditions of high load the ASC group demonstrates better ability to detect changes because they have more perceptual resources available to them than controls. Since detecting changes to the central interest items is easier than those to marginal interest items, this view predicts that the groups should not differ in the ability to detect central interest changes and that the ASC group should perform better than controls in detecting the marginal interest changes, which was what was found in the present study.

The present findings challenge results from some previous studies of change blindness in ASC that have reported no differences between groups or even greater change blindness in ASC [29–33] (Fletcher-Watson et al, 2006; Fletcher-Watson et al., 2008; Kikuchi et al., 2009; Loth et al., 2008; New et al., 2010). Mixed results across studies may be due to differences in methodologies and in characteristics of the samples [33,34] (Loth et al., 2008; Smith & Milne, 2008). For example, the task by Fletcher-Watson et al [29] (2006) utilized a ‘switch’ procedure which involved multiple response requirements throughout, which included pressing the space bar throughout each of the trials to progress from one image pair to the other, and then pressing further different buttons to stop the trial once the change was detected and to move to the next trial. Together, this may have increased task difficulty and EF demands compared to the traditional flicker paradigm and may have affected the results of that study, given the reported deficits of executive function in ASC [36,37] (Ozonoff, 1995; Ozonoff et al., 1991). A further change detection task [35] (Fletcher-Watson et al., 2012) and the present task both involved the same methodology where participants only had to press a button once during trials when they detected a change, which requires little cognitive demands during the task. Interestingly, both the present study and the one by Fletcher-Watson et al. [35] (2012) report about reduced change blindness in ASC to changes of marginal interest items, showing that methodology might be an important factor in studies of change detection in ASC.

The present study also found the greatest groups differences in detecting changes with location, where an item moved from one part of the scene to another across image alternations. This finding must be interpreted with caution for two main reasons. The first is that the groups also showed differences in the number of detected changes for the location types of changes, with the ASC group detecting approximately one fewer change compared to the controls. The second reason is that the images were not equated for detection difficulty across the different categories. However, it is still an interesting result for a couple of reasons. One reason is because changes of location often involve a wider area of the image compared to the other types of changes, as the change across images includes the original location of the scene where the image occurred along with the new location where the item appears in the changed image. In contrast, changes of colour and absence/presence involve changes occurring within the exact consistent location across images. So on the one hand, enhanced attention to detail may facilitate attention to changes of location in ASC because evidence of a change may appear across a wider area of the image compared to other types of changes, with some visual detail perhaps capturing the attention of those with ASC more readily than controls in most instances.

Another interesting reason is that many of the changes in location to marginal interest items occur in relation to items within the image that are of central interest (e.g. a bar that runs behind the two central people eating dinner in a scene and moves up and down across the two versions of the image). Therefore, detection of some changes in location within images may involve attention to relations between the marginal and central interest items, or shifting attention between the central and marginal items as the images change. Since those with ASC are reported to have difficulties in shifting attention [56,57] (Courchesne et al., 1994; Pascualvaca et al., 1998), widening their focus of attention [58] (Burack, 1994), or having an overly focused attention [15–17] (Robertson et al., 2013a,b,c), they may miss changes of location within certain scenes where relations to wider areas or aspects of the image in relation to the changing item are helpful to detect the change. This may help explain why the ASC group also had lower detection rates compared to the control group for changes to items of marginal interest that changed their location within the images, reflecting greater change blindness for detecting these types of marginal interest changes. This group difference reflected approximately 1 more change missed for the ASC group versus the controls for marginal interest changes of location, though there was no overall group difference in change detection in the study. This small but significant effect of greater change blindness in detection rates for location changes may be due to the ASC group having more difficulty on certain trials involving a change of location to marginal interest items where shifting attention or widening their focus of attention was a useful strategy. Further research to disentangle the factors determining when those with ASC show reduced change blindness versus greater change blindness is needed.

The longer latencies overall to detect marginal interest compared to central interest changes by both groups reveals a normal change blindness effect in ASC. This demonstrates that some degree of context or semantics about the information in the scenes was influencing attention processes in those with ASC. If context had no effect, we would have expected to see equivalent response times across both central and marginal changes in ASC, as their attention would be drawn to the physical characteristics of the changes and not influenced by the semantic aspects of the scene. The types of change and the low-level visual characteristics were well-matched between central and marginal interest conditions, therefore some degree of top-down influence was likely affecting attention in ASC. However, the present results show that attention in ASC is drawn more towards items of less interest in the scenes than the controls, suggesting that context had less effect in those with ASC than controls and that there may be differences in the nature of top-down influence for those with ASC. It should be noted that what counts as items of “more” or “less” interest itself depends on coding information for “relevance” [59] (Sperber, 1995), an ability linked to ‘theory of mind’ since a typical child or adult is constantly monitoring what is of social relevance to others. Thus, the current findings, whilst perceptual, may themselves be influenced by atypical social cognition in ASC [60] (Baron-Cohen et al., 2013).

A limitation of the present study is the use of longer latencies for displays compared to timings used in other studies of change blindness. We included the longer latencies because previous research has reported comparable change blindness effects are evident across both shorter and longer display times, including latencies similar to those used in the present study [3,4,41,43] (O’Regan et al., 1999; O’Regan et al., 2000; Rensink, 2000; Rensink et al., 2000), and because people with ASC often show slower responses compared to controls and have more difficulties with EF and decision-making. Although having longer latencies might help simplify the task and reduce the cognitive demands for those with ASC, it might also affect how they view the images and detect the changes in a way to favor those with ASC. However, another change detection study reported similar findings to the present study using shorter display timings of the images [35] (Fletcher-Watson et al., 2012). Further research is needed comparing different methodologies to test the effects of these factors on change detection in ASC.

Another limitation is that the present study included only male adults with ASC and participants generally had high IQ scores, so these results about reduced change blindness items of marginal interest may not generalize to other people with ASC. Therefore, further research is needed to test whether these results would be seen for other groups on the autism spectrum, including females and lower-functioning individuals. Future studies should incorporate other measures of gaze during the task, such as eye-tracking, to investigate overt attention by people with and without ASC. We conclude that we have found evidence for reduced change blindness in autism which represents superior change detection for items within scenes that are typically less salient.

## Conclusions

The present study is the first experimental evidence we are aware of to demonstrate reduced change blindness in adults with ASC for changes occurring in scenes of everyday life. The enhanced detection of changes in ASC occurred for items within scenes that are normally of less interest for people to attend towards and subsequently remember. However, the ASC group also showed a small but significant reduction in detecting changes of location within the marginal interest condition compared to the controls. Together, these results are consistent with theories of enhanced perception of local details alongside reduced processing of the wider context in ASC, and with clinical and anecdotal reports that people with ASC show differences in attention towards small details in the environment that often appear insignificant to others.

## Supporting information

S1 File Study data. This is the data that supports the results of the study.
(XLSX)
